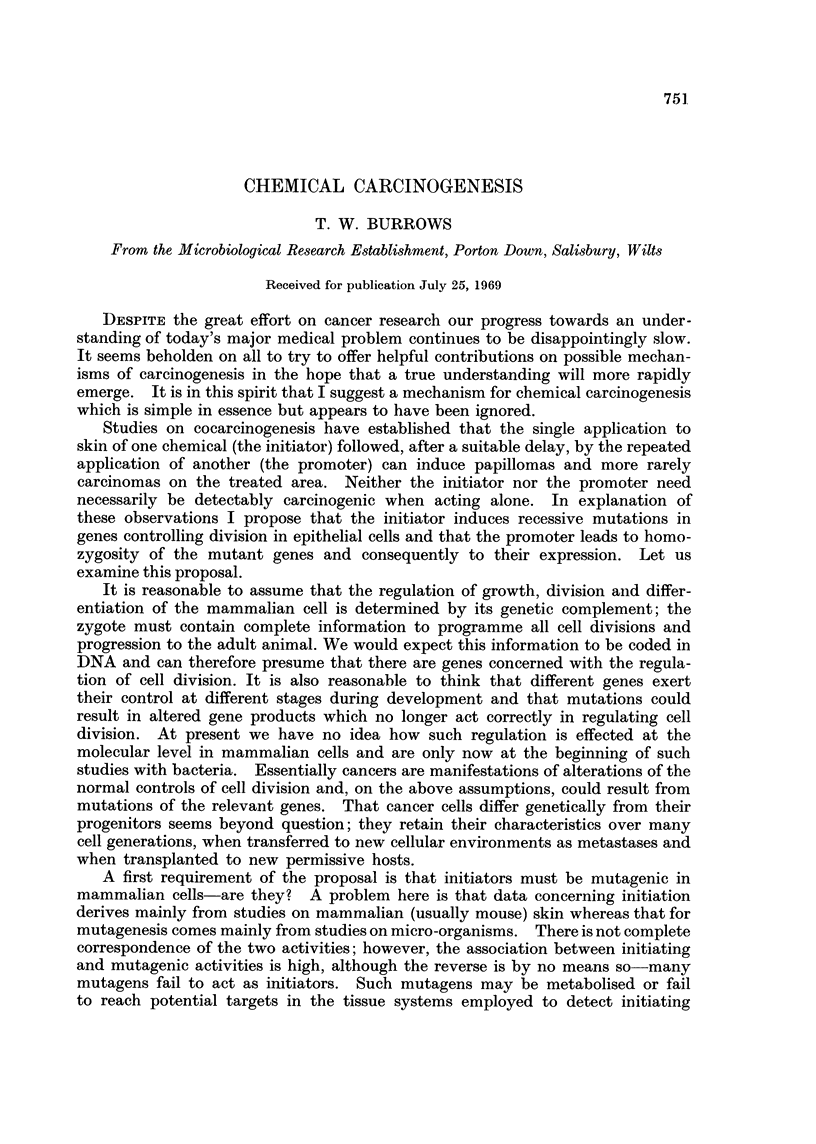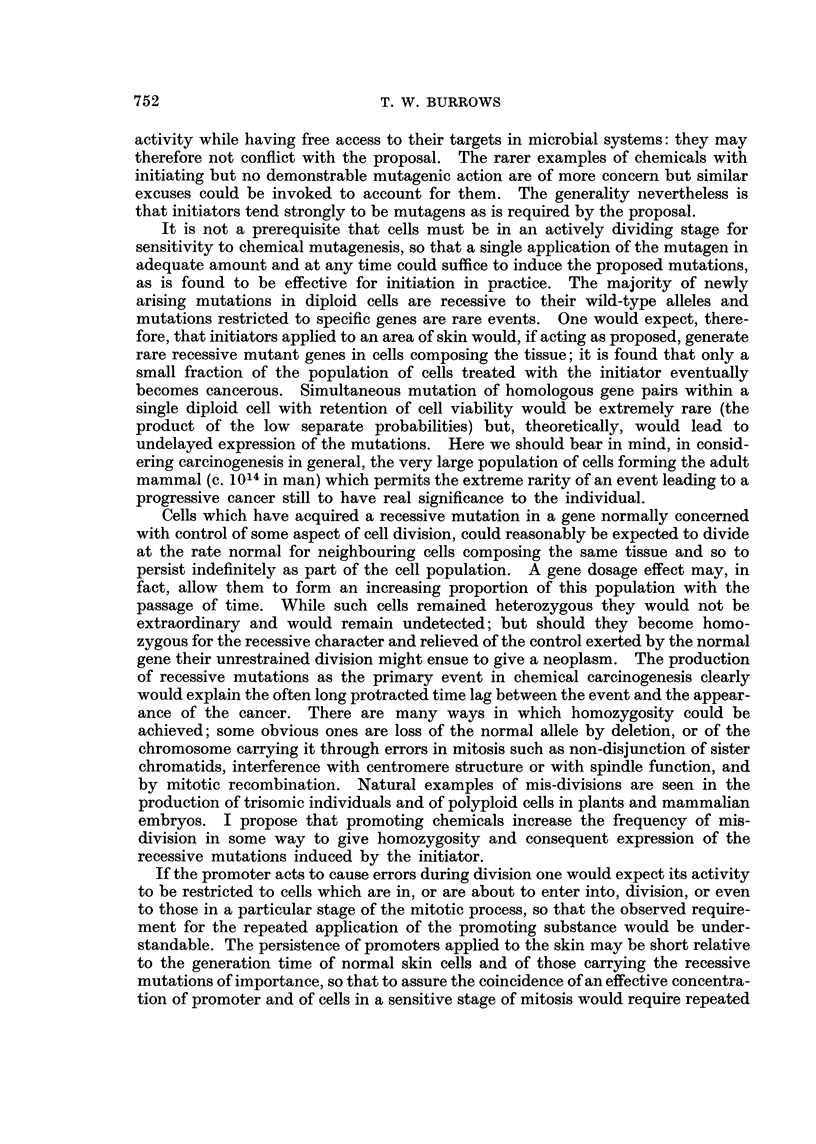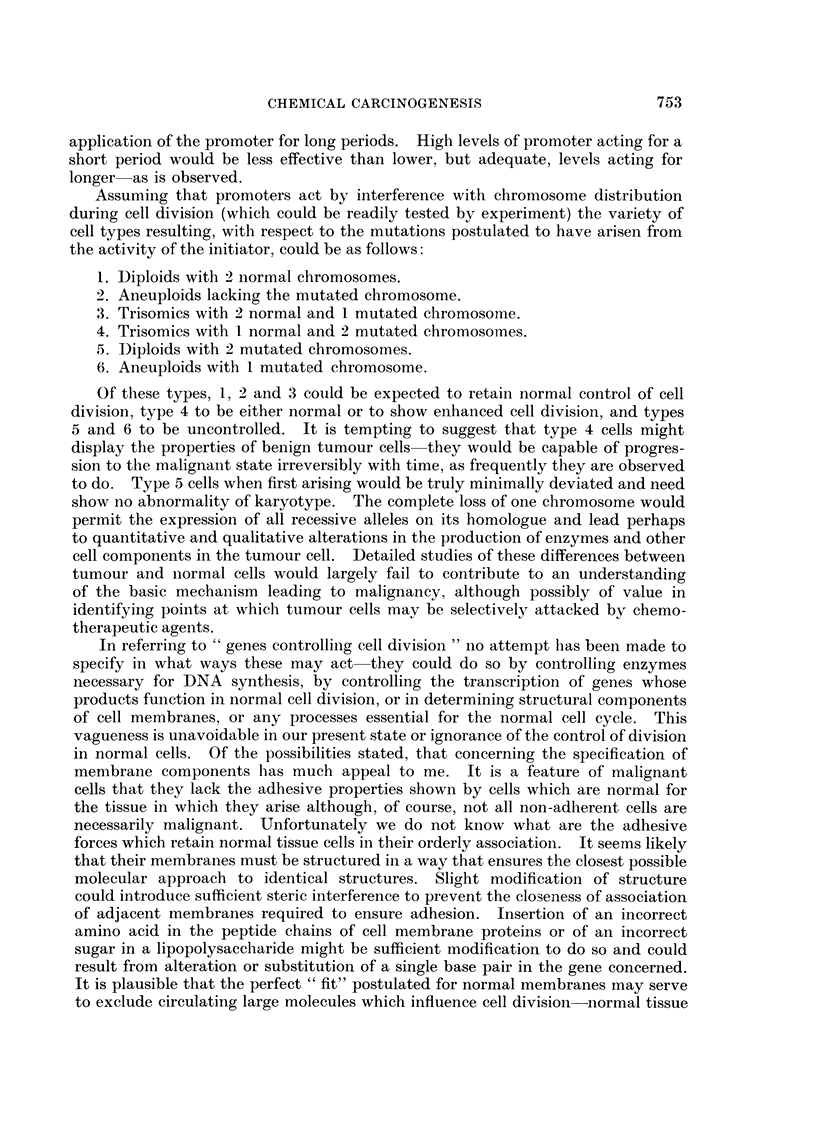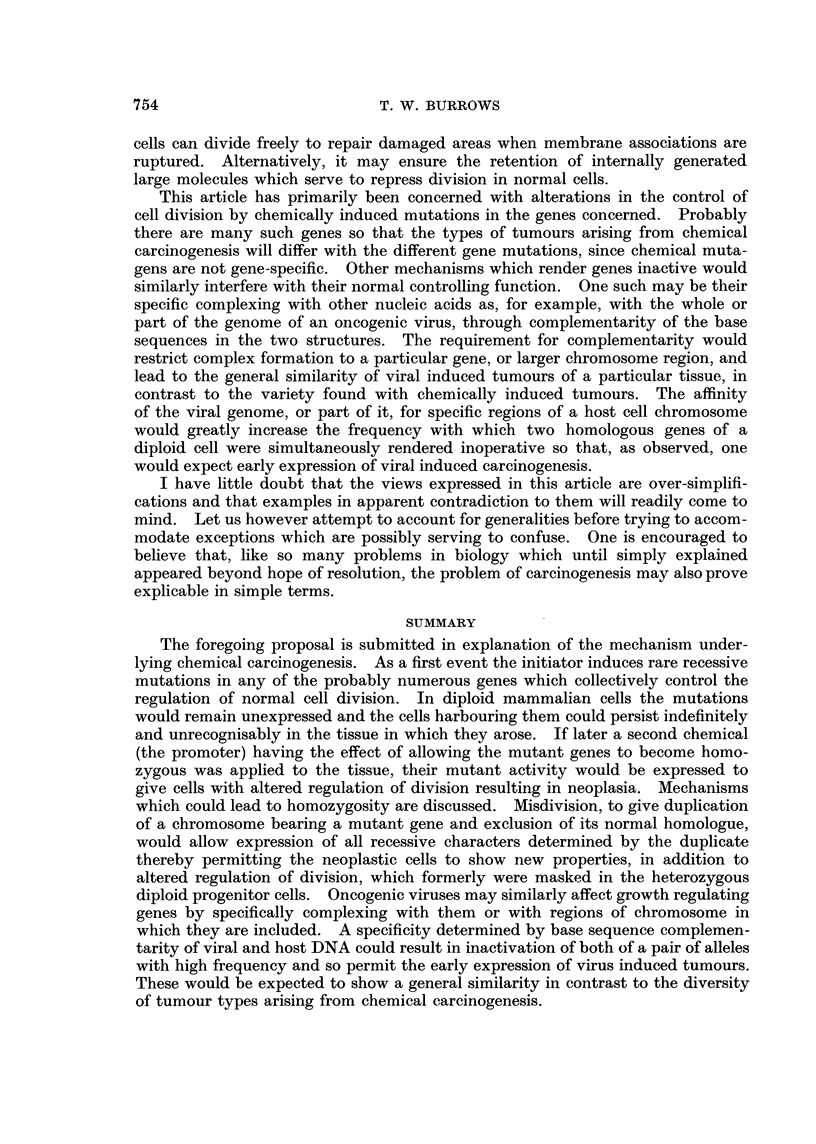# Chemical carcinogenesis.

**DOI:** 10.1038/bjc.1969.92

**Published:** 1969-12

**Authors:** T. W. Burrows


### CHEMICAL CARCINOGENESIS

T. W. BURROWS

*From the Microbiological Research Establishment, Porton Down, Salisbury, Wilts*

Received for publication July 25, 1969

DESPITE the great effort on cancer research our progress towards an understanding of today's major medical problem continues to be disappointingly slow. It seems beholden on all to try to offer helpful contributions on possible mechanisms of carcinogenesis in the hope that a true understanding will more rapidly emerge. It is in this spirit that I suggest a mechanism for chemical carcinogenesis which is simple in essence but appears to have been ignored.

Studies on cocarcinogenesis have established that the single application to skin of one chemical (the initiator) followed, after a suitable delay, by the repeated application of another (the promoter) can induce papillomas and more rarely carcinomas on the treated area. Neither the initiator nor the promoter need necessarily be detectably carcinogenic when acting alone. In explanation of these observations I propose that the initiator induces recessive mutations in genes controlling division in epithelial cells and that the promoter leads to homozygosity of the mutant genes and consequently to their expression. Let us examine this proposal.

It is reasonable to assume that the regulation of growth, division and differentiation of the mammalian cell is determined by its genetic complement; the zygote must contain complete information to programme all cell divisions and progression to the adult animal. We would expect this information to be coded in DNA and can therefore presume that there are genes concerned with the regulation of cell division. It is also reasonable to think that different genes exert their control at different stages during development and that mutations could result in altered gene products which no longer act correctly in regulating cell division. At present we have no idea how such regulation is effected at the molecular level in mammalian cells and are only now at the beginning of such studies with bacteria. Essentially cancers are manifestations of alterations of the normal controls of cell division and, on the above assumptions, could result from mutations of the relevant genes. That cancer cells differ genetically from their progenitors seems beyond question; they retain their characteristics over many cell generations, when transferred to new cellular environments as metastases and when transplanted to new permissive hosts.

A first requirement of the proposal is that initiators must be mutagenic in mammalian cells—are they? A problem here is that data concerning initiation derives mainly from studies on mammalian (usually mouse) skin whereas that for mutagenesis comes mainly from studies on micro-organisms. There is not complete correspondence of the two activities; however, the association between initiating and mutagenic activities is high, although the reverse is by no means so—many mutagens fail to act as initiators. Such mutagens may be metabolised or fail to reach potential targets in the tissue systems employed to detect initiating activity while having free access to their targets in microbial systems: they may therefore not conflict with the proposal. The rarer examples of chemicals with initiating but no demonstrable mutagenic action are of more concern but similar excuses could be invoked to account for them. The generality nevertheless is that initiators tend strongly to be mutagens as is required by the proposal.

It is not a prerequisite that cells must be in an actively dividing stage for sensitivity to chemical mutagenesis, so that a single application of the mutagen in adequate amount and at any time could suffice to induce the proposed mutations, as is found to be effective for initiation in practice. The majority of newly arising mutations in diploid cells are recessive to their wild-type alleles and mutations restricted to specific genes are rare events. One would expect, therefore, that initiators applied to an area of skin would, if acting as proposed, generate rare recessive mutant genes in cells composing the tissue; it is found that only a small fraction of the population of cells treated with the initiator eventually becomes cancerous. Simultaneous mutation of homologous gene pairs within a single diploid cell with retention of cell viability would be extremely rare (the product of the low separate probabilities) but, theoretically, would lead to undelayed expression of the mutations. Here we should bear in mind, in considering carcinogenesis in general, the very large population of cells forming the adult mammal (c. $10^{14}$ in man) which permits the extreme rarity of an event leading to a progressive cancer still to have real significance to the individual.

Cells which have acquired a recessive mutation in a gene normally concerned with control of some aspect of cell division, could reasonably be expected to divide at the rate normal for neighbouring cells composing the same tissue and so to persist indefinitely as part of the cell population. A gene dosage effect may, in fact, allow them to form an increasing proportion of this population with the passage of time. While such cells remained heterozygous they would not be extraordinary and would remain undetected; but should they become homozygous for the recessive character and relieved of the control exerted by the normal gene their unrestrained division might ensue to give a neoplasm. The production of recessive mutations as the primary event in chemical carcinogenesis clearly would explain the often long protracted time lag between the event and the appearance of the cancer. There are many ways in which homozygosity could be achieved; some obvious ones are loss of the normal allele by deletion, or of the chromosome carrying it through errors in mitosis such as non-disjunction of sister chromatids, interference with centromere structure or with spindle function, and by mitotic recombination. Natural examples of mis-divisions are seen in the production of trisomic individuals and of polyploid cells in plants and mammalian embryos. I propose that promoting chemicals increase the frequency of mis-division in some way to give homozygosity and consequent expression of the recessive mutations induced by the initiator.

If the promoter acts to cause errors during division one would expect its activity to be restricted to cells which are in, or are about to enter into, division, or even to those in a particular stage of the mitotic process, so that the observed requirement for the repeated application of the promoting substance would be understandable. The persistence of promoters applied to the skin may be short relative to the generation time of normal skin cells and of those carrying the recessive mutations of importance, so that to assure the coincidence of an effective concentration of promoter and of cells in a sensitive stage of mitosis would require repeated application of the promoter for long periods. High levels of promoter acting for a short period would be less effective than lower, but adequate, levels acting for longer—as is observed.

Assuming that promoters act by interference with chromosome distribution during cell division (which could be readily tested by experiment) the variety of cell types resulting, with respect to the mutations postulated to have arisen from the activity of the initiator, could be as follows:

1. Diploids with 2 normal chromosomes.
2. Aneuploids lacking the mutated chromosome.
3. Trisomics with 2 normal and 1 mutated chromosome.
4. Trisomics with 1 normal and 2 mutated chromosomes.
5. Diploids with 2 mutated chromosomes.
6. Aneuploids with 1 mutated chromosome.

Of these types, 1, 2 and 3 could be expected to retain normal control of cell division, type 4 to be either normal or to show enhanced cell division, and types 5 and 6 to be uncontrolled. It is tempting to suggest that type 4 cells might display the properties of benign tumour cells—they would be capable of progression to the malignant state irreversibly with time, as frequently they are observed to do. Type 5 cells when first arising would be truly minimally deviated and need show no abnormality of karyotype. The complete loss of one chromosome would permit the expression of all recessive alleles on its homologue and lead perhaps to quantitative and qualitative alterations in the production of enzymes and other cell components in the tumour cell. Detailed studies of these differences between tumour and normal cells would largely fail to contribute to an understanding of the basic mechanism leading to malignancy, although possibly of value in identifying points at which tumour cells may be selectively attacked by chemotherapeutic agents.

In referring to " genes controlling cell division " no attempt has been made to specify in what ways these may act—they could do so by controlling enzymes necessary for DNA synthesis, by controlling the transcription of genes whose products function in normal cell division, or in determining structural components of cell membranes, or any processes essential for the normal cell cycle. This vagueness is unavoidable in our present state or ignorance of the control of division in normal cells. Of the possibilities stated, that concerning the specification of membrane components has much appeal to me. It is a feature of malignant cells that they lack the adhesive properties shown by cells which are normal for the tissue in which they arise although, of course, not all non-adherent cells are necessarily malignant. Unfortunately we do not know what are the adhesive forces which retain normal tissue cells in their orderly association. It seems likely that their membranes must be structured in a way that ensures the closest possible molecular approach to identical structures. Slight modification of structure could introduce sufficient steric interference to prevent the closeness of association of adjacent membranes required to ensure adhesion. Insertion of an incorrect amino acid in the peptide chains of cell membrane proteins or of an incorrect sugar in a lipopolysaccharide might be sufficient modification to do so and could result from alteration or substitution of a single base pair in the gene concerned. It is plausible that the perfect " fit" postulated for normal membranes may serve to exclude circulating large molecules which influence cell division—normal tissue cells can divide freely to repair damaged areas when membrane associations are ruptured. Alternatively, it may ensure the retention of internally generated large molecules which serve to repress division in normal cells.

This article has primarily been concerned with alterations in the control of cell division by chemically induced mutations in the genes concerned. Probably there are many such genes so that the types of tumours arising from chemical carcinogenesis will differ with the different gene mutations, since chemical mutagens are not gene-specific. Other mechanisms which render genes inactive would similarly interfere with their normal controlling function. One such may be their specific complexing with other nucleic acids as, for example, with the whole or part of the genome of an oncogenic virus, through complementarity of the base sequences in the two structures. The requirement for complementarity would restrict complex formation to a particular gene, or larger chromosome region, and lead to the general similarity of viral induced tumours of a particular tissue, in contrast to the variety found with chemically induced tumours. The affinity of the viral genome, or part of it, for specific regions of a host cell chromosome would greatly increase the frequency with which two homologous genes of a diploid cell were simultaneously rendered inoperative so that, as observed, one would expect early expression of viral induced carcinogenesis.

I have little doubt that the views expressed in this article are over-simplifications and that examples in apparent contradiction to them will readily come to mind. Let us however attempt to account for generalities before trying to accommodate exceptions which are possibly serving to confuse. One is encouraged to believe that, like so many problems in biology which until simply explained appeared beyond hope of resolution, the problem of carcinogenesis may also prove explicable in simple terms.

### SUMMARY

The foregoing proposal is submitted in explanation of the mechanism underlying chemical carcinogenesis. As a first event the initiator induces rare recessive mutations in any of the probably numerous genes which collectively control the regulation of normal cell division. In diploid mammalian cells the mutations would remain unexpressed and the cells harbouring them could persist indefinitely and unrecognisably in the tissue in which they arose. If later a second chemical (the promoter) having the effect of allowing the mutant genes to become homozygous was applied to the tissue, their mutant activity would be expressed to give cells with altered regulation of division resulting in neoplasia. Mechanisms which could lead to homozygosity are discussed. Misdivision, to give duplication of a chromosome bearing a mutant gene and exclusion of its normal homologue, would allow expression of all recessive characters determined by the duplicate thereby permitting the neoplastic cells to show new properties, in addition to altered regulation of division, which formerly were masked in the heterozygous diploid progenitor cells. Oncogenic viruses may similarly affect growth regulating genes by specifically complexing with them or with regions of chromosome in which they are included. A specificity determined by base sequence complementarity of viral and host DNA could result in inactivation of both of a pair of alleles with high frequency and so permit the early expression of virus induced tumours. These would be expected to show a general similarity in contrast to the diversity of tumour types arising from chemical carcinogenesis.